# The Roles of Distinct Transcriptional Factors in the Innate Immunity of *C. elegans*

**DOI:** 10.3390/cells14050327

**Published:** 2025-02-21

**Authors:** Muhammad Irfan Afridi, Haijun Tu

**Affiliations:** 1 State Key Laboratory of Chemo/Biosensing and Chemometrics, College of Biology, Hunan University, Changsha 410082, China; irfan.afridi@hnu.edu.cn; 2 Shenzhen Research Institute, Hunan University, Shenzhen 518000, China

**Keywords:** host–pathogen interaction, transcription factors, WH-FORKHEAD TFs, bZIP TFs, GATA TFs, innate immunity

## Abstract

Deleterious molecules or factors produced by pathogens can hinder the normal physiological functioning of organisms. In response to these survival challenges, organisms rely on innate immune signaling as their first line of defense, which regulates immune-responsive genes and antimicrobial peptides to protect against pathogenic infections. These genes are under the control of transcription factors, which are known to regulate the transcriptional activity of genes after binding to their regulatory sequences. Previous studies have employed *Caenorhabditis elegans* as a host–pathogen interaction model to demonstrate the essential role of different transcription factors in the innate immunity of worms. In this review, we summarize the advances made regarding the functioning of distinct transcription factors in the innate immune response upon pathogen infection. Finally, we discuss the open questions in the field, whose resolutions have the potential to expand our understanding of the mechanisms underlying the innate immunity of organisms.

## 1. Introduction

All organisms living in the environment face the challenge of being colonized and invaded by different pathogens. Consequently, all forms of life have developed mechanisms to maintain homeostasis, seeking to prevent tissue damage and destroy pathogens [1]. The immune system represents a typical tool used to counteract pathogenic invasion for survival through innate and adaptive immunity [2]. The innate immune response is rapid and non-specific, and in contrast, the adaptive immune response is slow but more specific [3]. Generally, innate immunity is evolutionarily conserved across species, while adaptive immunity has evolved in higher organisms such as vertebrates [4]. The initial line of defense against pathogens comprises physical barriers that are responsible for blocking pathogen entry [5]. The failure of this mechanism results in the activation of the innate immune response, which employs pattern recognition receptors (PRRs) to recognize a broad range of pathogen-associated molecular patterns (PAMPs). Upon recognition, these PRRs induce signaling pathways, including transcription factors (TFs), to activate immune response molecules [6,7].

In mammals, TFs such as nuclear factor kappa B (NF-κB) and the AP-1 and STAT proteins play a crucial role in mediating the immune system through the regulation of transcripts involved in cell survival, inflammation, and pathogen defense [8,9]. NF-κB, associated with the inflammatory response, is induced by different stimuli, which include pathogens and stress signals [9]. The STAT1 and STAT3 proteins are crucial components of cytokine signaling that modulate the immune response by inducing or blocking specific target genes’ expression [10]. AP-1, composed of c-Jun, c-Fos, and related proteins, is necessary for gene activity to activate immune cells and enable differentiation [11]. These regulatory mechanisms underscore the importance of TFs in the immune response. The nematode *Caenorhabditis elegans* (*C. elegans*) provides a suitable context to study these evolutionarily conserved mechanisms, as the TF families in worms share 37% of their gene orthologs with humans [12,13].

The free-living, soil-dwelling nematode *C. elegans* has several features that make it a suitable model organism to study host–pathogen interactions. Due to its small size, *C. elegans* is easy to handle in the laboratory. It is transparent, self-fertilizing, and develops from an egg to a gravid adult in three days at 22 °C, with a total lifespan of approximately 3–5 weeks [14]. The worms are naturally bacterivorous and harbor unique microbiota [15]. Importantly, many human pathogens (bacteria and fungi) that have been shown to infect *C. elegans* ultimately lead to its death (Table 1) [16,17,18]. This feature can be utilized in the laboratory to infect worms, replacing their laboratory food (non-pathogenic *Escherichia coli* strain OP50) with a pathogen of interest [19]. In *C. elegans*, infection can occur through different entry sites, such as the cuticle, epidermis, or rectum, leading to subsequent colonization in the intestine [17,20,21]. However, this route is not necessarily applicable to all pathogens, especially those that infect the epidermis [22]. *Pseudomonas aeruginosa* is an opportunistic, Gram-negative pathogen that infects and affects both mammals and worms in a similar manner. This supports the use of *P. aeruginosa* to infect *C. elegans* as a suitable model organism for the study of host–pathogen interactions [20]. Moreover, virulence factors of *Salmonella enterica*, including serovars such as *S. typhimurium*, have been reported to cause infection and lead to death in worms [17]. These findings have paved the way for the exploration of a range of pathogens that infect *C. elegans*, making this a powerful genetic model to study bacterial pathogenesis mechanisms that are applicable to mammals [22,23,24,25,26,27]. In response to pathogens, *C. elegans* adopts two distinct strategies for survival: either avoiding the pathogenic environment through behavioral changes [28,29] or triggering molecular signaling pathways that are associated with the immune response, such as P38/PMK-1 [30], DBL-1/TGF-β [31], and FSHR-1 [32]. Here, we highlight some of the pathogens that infects humans and induces an immune response in *C. elegans* (Table 1). 

Pathogens that induce innate immunity in *C. elegans* trigger the expression of various genes, including TFs, in response (Table 2) [37]. The expression patterns of several immune response genes are dependent on specific TFs. However, the TFs of *C. elegans*, such as DAF-16 (DAF-2/DAF-16) [38], ZIP-2 (ZIP-2/IRG-1) [39], and ELT-2 (ELT-2/GATA) [40], regulate distinct sets of immune-specific target genes and are solely responsible for the regulation of immune pathways, being independent of other known immune pathways such as P38/PMK-1 [30], DBL-1/TGF-β [31], and FSHR-1 [32]. Additionally, several TFs have been reported to be essential in previously recognized immune signaling pathways. Although *C. elegans* contains a large number of transcription factors [12], only members of WH-FORKHEAD, bZIP, and GATA have been extensively recognized for their roles in mediating the immune response. Meanwhile, the involvement of other transcription factor families in the immune response remains largely unexplored. In this review, we address the gaps in our understanding of these TFs, such as DAF-16 (WH-FORKHEAD), ZIP-2, SKN-1, ATF-7 (bZIP), and ELT-2 (GATA) TFs. We focus on ATFS-1, CEBP-2, and some members of the nuclear hormone receptor, homeobox, and basic helix–loop–helix TF families in regulating the innate immunity of *C. elegans*. We also discuss the research directions that could further advance our knowledge of the TF-mediated immune response.

## 2. Winged Helix Forkhead TF

### 2.1. Overview of Forkhead TFs

**Figure 1:**
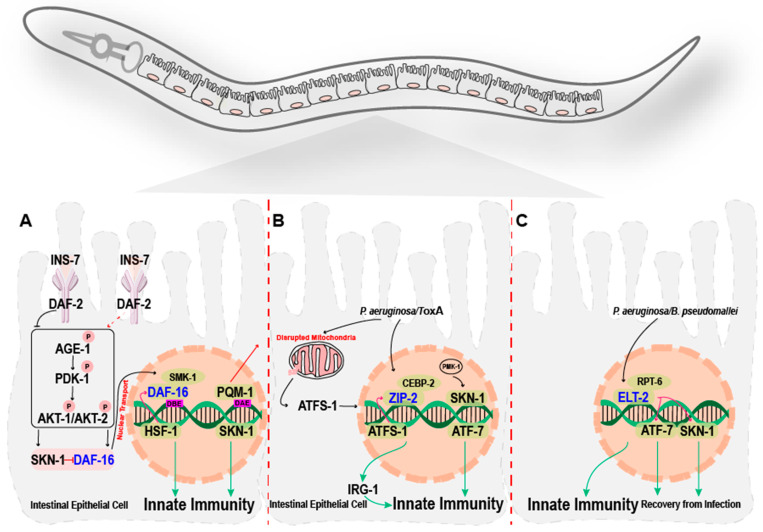
Members of the WH-FORKHEAD, bZIP, and GATA transcription factor families are required to mediate immune responses in *C. elegans*. (**A**–**C**): DAF-16 (**A**), ZIP-2 (**B**), and ELT-2 (**C**), as central members of the WH-FORKHEAD, bZIP, and GATA families, respectively, which regulate the immune response individually and in coordination with other transcripts.

The Forkhead (FOX) gene family is evolutionarily conserved from yeast to humans, incorporating various subfamilies of TFs that share a winged helix DNA-binding domain. Winged helix FOX TFs play an important role in development, metabolism, aging, cancer, and immunity [44,45,46]. Consequently, the dysregulation of the FOX genes leads to impaired biological functioning including immunological defects [46]. The FOXO proteins are vital transcriptional effectors of the evolutionarily conserved insulin/IGF-1 signaling (IIS) pathway, which is known to regulate longevity [38,47]. The FOXO proteins are a subfamily of the Forkhead family of TFs. They are conserved from *C. elegans* to mammals; *C. elegans* has one FOXO protein encoded by the *daf-16* gene, and mammals have four FOXO proteins encoded by several genes, i.e., FOXO1 (FKHR), FOXO3 (FKHRL1), FOXO4 (AFX), and FOXO6 [48,49]. Extensive studies on *C. elegans* have revealed the role of the FOXO proteins in longevity through their interaction with the IIS pathway, linking DAF-2 (a homolog of the insulin receptor) and its downstream effector DAF-16 (a homolog of FOXO) [49,50]. In *C. elegans*, the loss of function of *daf-2* significantly increases the organism’s longevity, improves its health, and provides protection from age-related diseases [51]. Under normal conditions, appropriate stimulus binding to the transmembrane DAF-2/insulin receptor activates the IIS pathway, which in turn triggers the AKT (protein kinase B)-dependent suppression of the phosphorylation and nuclear translocation of DAF-16/FOXO [52]. Under stress conditions, the reduced expression of DAF-2/insulin receptors alleviates the repression of DAF-16 phosphorylation, which allows for the activation of the expression of target genes after binding to their target consensus DNA motif TTGTTTAC via the DAF-16-binding element (DBE) (Table 3) [53]. This regulatory mechanism is shared between mammals and *C. elegans* (Figure 1A).

The extension of *C. elegans*’ lifespan is associated with the repression of the IIS pathway, particularly in *daf-2* mutants. This repression leads to the upregulation of stress response genes (class 1) and downregulation of other genes (class 2) that are specifically dependent on the functioning of DAF-16 [54]. Notably, DAF-16 directly regulates class 1 genes by binding to the conserved DBE regions of key genes such as *sod-3* and *mtl-1* [54,55]. These class 1 genes are associated with stress resistance, detoxification, and cellular maintenance, which collectively contribute to longevity in *daf-2* mutants. However, the class 2 genes associated with metabolic processes are downregulated in *daf-2* mutants and upregulated in *daf-16* (RNAi), which indicates an antagonistic role in the regulation of longevity [54]. Additionally, the DAF-16-associated element (DAE) DNA motif has been identified, which is bound by another TF, PQM-1 (controlling class 2 developmental gene expression), to complement DAF-16’s role in longevity [56]. Under an active state of IIS, DAF-16 is inactive and PQM-1 promotes growth and development. Under reduced IIS, PQM-1 exits the nucleus, which allows DAF-16 to translocate to the nucleus and activate stress-responsive genes [56]. In conclusion, nuclear-accumulated intestinal DAF-16 regulates the transcription of several genes that participate in promoting stress resistance, metabolizing fat, protecting against pathogens, and regulating dauer formation and lifespan in *C. elegans* [49].

### 2.2. Immune Pathways Regulated by DAF-16

The DAF-16-mediated immune pathway is independent of the evolutionarily conserved p38 MAPK or the DBL-1/TGF-β pathway [31]. Loss-of-function mutations in *daf-2* and *age-1* enhance resistance to pathogenic bacteria by promoting the phosphorylation and nuclear translocation of *daf-16*, upregulating the expression of DAF-16-dependent genes. Despite this, the post-infection survival rate of *daf-16* mutants is comparable to that of wild-type worms [38,57]. However, a tissue-specific study has highlighted that DAF-16 can be triggered in the epidermis in response to fungal infections, independently of the DAF-2 pathway [58]. A *daf-16* mutation suppresses the resistance of *daf-2* mutant worms against pathogens, indicating that *daf-2* mutants’ resistance relies on active DAF-16 [57]. In conclusion, the DAF-2–DAF-16 immune signaling pathway regulates the expression of immune response genes against different pathogens [54,57,59,60]. Moreover, pathogen infection increases the expression of insulin-like peptide INS-7, an upstream molecule of DAF-2, and activates insulin signaling, which will inhibit DAF-16 and downregulate its target immune response genes [60]. This suggests that insulin signaling and neuroendocrine pathways, including those involving INS-7, influence DAF-6’s activity in response to pathogen stress (Figure 1A).

#### Interactions with Other TFs

The heat shock TF (HSF-1) enhances immunity in *C. elegans* and is involved in the DAF-2/DAF-16 signaling pathway [61,62]. The expression of HSF-1 proteins is induced in overexpressed *daf-16* and *daf-2* mutants in response to pathogens, suggesting its necessity for the DAF-2–DAF-16 signaling pathway in this context (Figure 1A) [61]. In terms of longevity, DAF-16’s activity is complemented by that of SMK-1. SMK-1 is an essential nuclear co-regulator of DAF-16; it has no defined role in dauer formation or in the reproductive function of DAF-16 [63]. Worms deficient in *daf-2* activity exhibit pathogenic and non-pathogenic responses, which are closely associated with the IIS pathway with the response depending on SMK-1 activity. This indicates that the activity of SMK-1 is crucial in regulating DAF-16-mediated immunity (Figure 1A) [63,64].

The expression levels of immune effectors such as PMK-1/p38 MAP kinase are diminished over the lifespan, while the DAF-2–DAF-16 immune pathway continuously protects *C. elegans*, even in the absence of stress [64]. Both PMK-1/p38 MAP kinase and IIS regulate the SKN-1 TF, which is responsible for oxidative and xenobiotic stress responses in worms [65,66]. Activated IIS counteracts DAF-16, along with SKN-1, in parallel. A reduction in IIS helps to increase *C. elegans*’ resistance to oxidative stress and its longevity due to the accumulation of SKN-1 TFs in the nucleus [66]. This demonstrates that DAF-2 inhibits SKN-1 in parallel with DAF-16 (Figure 1A). Moreover, SKN-1 has been found to inhibit the nuclear transport of DAF-16 and downregulate the expression of DAF-16 target genes under mitochondrial, oxidative, and heat stress [67].

### 2.3. Potential Areas for Future Research

Several studies have highlighted the roles of DAF-16 in regulating immunity, either independently or through interactions with other TFs in the immune system. However, there are still several gaps in our understanding of DAF-16’s role. For instance, DAF-16 is activated in several tissue types, but how it regulates the immune response in distinct tissue types and the tissues crosstalk through DAF-16 remains underexplored. Moreover, as discussed above, SKM-1 is known for its interaction with DAF-16, but how SMK-1 interacts with HSF-1 remain unclear. While several pathogens have been reported to cause infection in *C. elegans* (Table 1), it is unknown how DAF-16 influences different pathogens.

**Table 3:** TFs with their recognition sequences and their immune target genes.

TF	DNA-Binding Domain	Motif	Immune Target Genes	References
DAF-16	WH–Forkhead, AT Hook	5-TTGTTTAC-3′	e.g., *thn-2*, *lys-7*, *abf-2*, *sod-3*, *mtl-1*	[53,54,60,68]
ZIP-2	bZIP	ND	e.g., *irg-1*, *irg-2*, F11D11.3, *oac-32*	[39,69]
SKN-1	bZIP	5-ATGA-3′	e.g., *gcs-1*, *gst-4*, *gst-5*, *gst-7*	[17,66,70]
ATF-7	bZIP	5-ACGTCA-3′	e.g., T24B8.5, K08D8.5, C17H12.8	[71,72]
ELT-2	ZF–GATA	ND	e.g., F55G11.2, F08G5.6, *lys-2*	[40,73]

## 3. Basic Leucine Zipper Transcription Factors

### 3.1. Overview of bZIP TFs

Basic leucine zipper (bZIP) TFs are a family of conserved eukaryotic proteins that bind to specific regulatory DNA sequences as homodimers or heterodimers to activate or repress gene transcription [74,75]. These bZIP TFs share a conserved domain for basic DNA binding and a leucine zipper dimerization domain that is essential for their DNA-binding activity [74,76]. In *C. elegans*, there are 33 bZIP TF members, whereas humans possess approximately 55 bZIP TFs [12,77]. They act in networks and regulate a diverse set of cellular processes, including learning and memory, lipid metabolism, cancer progression, autophagy, and immunity [78,79,80]. Although numerous bZIP TF members play important roles in immune regulation [71,81,82], we mainly highlight three members of the bZIP TF family, namely ZIP-2, SKN-1, and ATF-7, due to their highly important roles in the innate immunity of *C. elegans*.

### 3.2. Immune Pathways Regulated by bZIP TFs

#### 3.2.1. ZIP-2

ZIP-2 is a specialized TF that regulates immune pathways in *C. elegans* independently of the immune pathways associated with *P. aeruginosa* infection, such as the PMK-1/p38 MAPK pathway [39]. ZIP-2 activity is necessary for *irg-1* (immune response gene) and the expression of several other immune transcripts [39]. The ZIP-2/IRG-1 immune pathway is triggered by exotoxin A (ToxA) in *P. aeruginosa* to inhibit host translation. An increase in the ZIP-2 protein level is triggered either directly through infection or via the release of toxins that are involved in translational inhibition (Figure 1B). Along with the ZIP-2/IRG-1 immune pathway, other specific immune response pathways associated with *P. aeruginosa* infection, such as PMK-1 and FSHR-1, are activated independently upon ToxA-mediated translational inhibition [69].

Mitochondrial stress impairs mitochondrial function, which allows the bZIP TF ATFS-1 to mediate the mitochondrial unfolded protein response (UPR) through the trafficking of ATFS-1 from the cytosol to the nucleus, which ultimately restores mitochondrial homeostasis [83]. The absence of *atfs-1* renders worms more susceptible to *P. aeruginosa* infection, and this was found to regulate the ZIP-2-dependent immune response. Intriguingly, mitochondrial stress induces the mRNA of *zip-2* and triggers IRG-1 expression. Alongside ZIP-2, the induction of IRG-1 under *P. aeruginosa* infection was found to be significantly diminished in the context of *atfs-1* mutants, suggesting that *atfs-1* can act upstream of ZIP-2 (Figure 1B) [84]. Moreover, CEBP-2 satisfies the criteria for being a potential heterodimeric partner of ZIP-2. CEBP-2, which is a bZIP TF and the *C. elegans* ortholog of mammalian C/EBP-gamma. CEBP-2 works together with ZIP-2 in the surveillance of immunity against *P. aeruginosa* [85]. CEBP-2/ZIP-2 not only acts against the intestinal translational block by *P. aeruginosa* ToxA (Figure 1B), but also interferes in other signaling processes, such as the histone and mitochondrial signaling pathways [85]. ZIP-2 also plays a central role in longevity and healthy aging under dietary restriction (DR) in *C. elegans* [86]. DR can significantly delay the aging process through translational inhibition, which triggers ZIP-2’s activity [86]. The role of ZIP-2 in aging is independent of its role in immunity.

#### 3.2.2. SKN-1

The Nrf2 family of transcription factors contributes to several crucial functions, such as stress response and metabolism [87,88]. In *C. elegans*, the functional and sequence ortholog of Nrf/CNC is SKN-1, a developmental specification protein [89,90]. Although the mode of binding to their target DNA is different between Nrf/CNC and SKN-1, they are functionally conserved, which indicates the importance of *C. elegans* SKN-1 in better understanding the mammalian Nrf2 and Nrf/CNC proteins. SKN-1 recognizes and binds with its target DNA in a unique mechanism, exhibiting high affinity as a monomer through the basic region, as seen in other bZIP proteins, but it lacks ZIP segments, which are essential for bZIP proteins’ DNA binding [91]. SKN-1’s role in oxidative stress has been extensively studied. Under optimal conditions, *C. elegans* SKN-1 is expressed in the nuclei of ASI chemosensory neurons and will accumulate in the intestine in response to oxidative stress. Worms lacking SKN-1 have shorter lifespans under oxidative stress [92].

SKN-1, which is a bZIP TF, mediates *C. elegans*’ immunity [70,93]. SKN-1 expression is induced in the *C. elegans* intestine during infection with *Enterococcus faecalis* or *P. aeruginosa*. Worms lacking SKN-1 have shown increased susceptibility to pathogens compared to worms with active SKN-1 [70]. The PMK-1/p38 immune pathway regulates the SKN-1 TF, not only under oxidative stress [65], but also during pathogen exposure (Figure 1B) [70]. Animals exposed to pathogens generate reactive oxygen species (ROS) as a defense mechanism [59]. During infection, dual oxidase CeDuox1/BLI-3 produces ROS, which subsequently activates PMK-1/p38. This activation triggers the phosphorylation and the nuclear localization of SKN-1 to resist pathogen infection [70]. Furthermore, the expression of SKN-1-dependent target genes is downregulated as worms age, suggesting that SKN-1 may play a role in immunosenescence. Functional SKN-1 is a prerequisite for survival beyond 9 days in *C. elegans* and in response to reduced IIS or preconditioning H2O2 treatment [93], linking stress response to an enhanced lifespan and delayed immunosenescence. Hyperglycemia can cause severe health complications [94,95]. This stress condition specifically affects the SKN-1-mediated innate immunity, rendering worms more susceptible to infection. Subsequently, upon the hyperactivation of SKN-1, the negative effect of glucose on SKN-1-regulated immunity is eliminated [96]. Moreover, exposure to zinc oxide nanoparticles has been found to hinder the intestinal nuclear localization of SKN-1, ultimately inhibiting SKN-1-mediated immunity [97]. Recent research indicates that its isoform, SKN-1A, is crucial to regulating tissue-specific proteasomal activity, which in turn modulates immune responses to different pathogens. This regulation ensures that immune activation is balanced and appropriately controlled, preventing hyperactivated or misdirected responses [98]. In conclusion, SKN-1 is an important immune regulator and is also affected by different environmental factors, which may reduce the level of innate immunity that it can confer.

#### 3.2.3. ATF-7

The innate immune response against pathogens is controlled by the phosphorylation of various targets of the PMK-1/p38 signaling pathway, including cyclic AMP-responsive element binding (CREB) and activating TF (ATF) families, such as ATF-2, which are also regulated by parallel pathways such as JNK, another member of the MAPK cascade [99,100,101]. In *C. elegans*, ATF-7, which is a bZIP TF and an assumed ortholog of mammalian ATF-2, acts downstream of the PMK-1/p38 MAPK-dependent immune pathways [72]. Under normal conditions, non-phosphorylated ATF-7 represses PMK-1/p38 MAPK-dependent immune response genes. However, ATF-7’s phosphorylation due to the induction of PMK-1/p38 activity after pathogen stress ultimately changes the mode of ATF-7 from a repressor to an activator of PMK-1/p38-dependent immune targets (Table 3, Figure 1B) [71,72]. Moreover, the drug rotenone causes mitochondrial dysfunction, requiring the ATF-7-PMK-1/p38 MAPK-dependent innate immune pathway in the intestine. This protects *C. elegans* against rotenone-induced dopaminergic neuronal loss due to mitochondrial loss [102]. However, the activation of ATF-7 by the TIR-dependent PMK-1/p38 MAPK immune pathway in response to the expression of mutant proteins associated with amyotrophic lateral sclerosis (ALS) in *C. elegans* motor neurons caused motor neuron degeneration [103], highlighting the context-dependent nature of ATF-7 in neuroprotection and neurodegeneration [102,103]. Recently, the olfactory neuronal gene *olrn-1*, known to control the development of AWC neurons, was found to regulate immunity in a cell-non-autonomous manner [104,105]. During larval development, the low activity of neuronal OLRN-1 in AWC chemosensory neurons boosts the ATF-7–PMK-1/p38 MAPK immune pathway in the intestine, indicating that there is an interplay between immunity and neuronal development. Overall, ATF-7 is an important immune regulator that works by integrating the immune system with neuronal development, neuroprotection, and neurodegeneration.

### 3.3. Potential Areas for Future Research

Although a significant amount of knowledge has been obtained regarding the roles of bZIP TFs, ZIP-2, SKN-1, and ATF-7 in the defense mechanisms of *C. elegans*, several areas remain unexplored. For instance, the role of ZIP-2 in regulating the immune response to a wider variety of pathogens has been poorly studied, despite its important role. Additionally, the linkage of ATF-7 with neural health, the relationships between numerous environmental factors that influence SKN-1’s activity, and the external conditions that induce innate immunity still need to be explored. Moreover, ATFS-1 and ATF-7 have been reported to play roles in the mitochondrial stress response, but these TFs’ interactions about mitochondrial and pathogen stress remain unclear.

## 4. GATA Transcription Factors

### 4.1. Overview of GATA TFs

GATA transcriptional family members share the consensus DNA sequence 5′-[AT]GATA[AG]-3′ and possess one or two zinc finger motifs with a highly conserved basic region, comprising a DNA-binding domain. They are conserved across vertebrates, invertebrates, fungi, and plants [106] and have been reported as vital factors in various developmental processes, such as cell fate specification and differentiation. The roles of GATA TFs in cell differentiation and tissue specification have been widely explored [107]. ZF-GATA TFs regulate the immunity of worms at the epidermal and intestinal levels against different types of stress [108]. These TFs regulate the immune response in the context of gut pathogens [40,73,109,110].

### 4.2. ELT-2 GATA TF

In young adult *C. elegans* exposed to *P. aeruginosa*, most immune response genes share a common GATA motif, which emphasizes the importance of GATA TFs as major regulators of intestinal genes [40]. ELT-2, which is a consensus sequence (A/T) GATA (A/G)-binding TF, was found to be significantly induced during PA14 infection, and its knockdown rendered worms more susceptible to pathogen infection [40]. ELT-2 is reportedly crucial for intestinal cell development. However, it also contributes to and remains active after development in adult worms to regulate innate immunity [40,111]. Furthermore, GATA6 is the human homolog of ELT-2; it protects lung epithelial cells from pathogen infection, which indicates the conserved role of GATA TFs in the response to pathogens [40]. Moreover, ELT-2 appears to be required for immune responses to multiple bacterial and fungal infections, demonstrating that ELT-2 may be an ancestral TF required for immune response, similarly to NF-kB TF in mammals [73,112]. ELT-2 also cooperates with the immune signaling pathway PMK-1/p38 and its phosphorylated TFs SKN-1 and ATF-7 in regulating *C. elegans* immunity (Figure 1C) [113].

Infection with *B. pseudomallei* strain R15 (BpR15) causes transcriptional changes in *C. elegans*. Initially, many genes are upregulated in response to BpR15 infection, but with prolonged exposure to the infection, the expression of these immune response genes becomes suppressed [110]. BpR15 also initially induces the expression of ELT-2 and its target genes, but the expression of ELT-2 target genes was also found to be repressed with increasing durations of infection. Surprisingly, the target genes of ELT-2 induced by PA14 were suppressed by BpR15, indicating that the immune responses of ELT-2 and its target genes differ upon infection with different pathogens (Figure 1C) [110]. The bacterial virulence factor T3SS can hijack and exploit the eukaryotic ubiquitin–proteasome system (UPS) for proteolysis [114,115,116]. The host UPS and BpR15 virulence factor T3SS (type III secretion system) were found to be necessary for ELT-2 protein loss; ultimately, BpR15 insult suppresses immunity through the downregulation of the ETL-2 response [110]. In an acute infection model of *C. elegans*–*S. enterica* pathogenesis, in the recovery phase, several cellular homeostatic pathways, such as xenobiotic detoxification, redox regulation, and cytoprotection are activated, while the immune response genes induced during infection are attenuated [73,117]. Genes with altered expression during the recovery phase of infection in the intestine are regulated by ELT-2. During recovery from acute infection, ELT-2’s functioning is vital, indicating its consistent role during and after infection [117]. This function of ELT-2 was supported by and further explored in the *C. elegans*–*P. aeruginosa* acute infection model, which highlighted the importance of ELT-2 and the p38-MAPK/PMK-1 immune pathway in the recovery phase during *P. aeruginosa* or *S. enterica* infection [118]. However, the role of ELT-2 is more obvious and crucial in the early phase of recovery compared to PMK-1/p38, which shows more prominent activity later. Furthermore, the DAF-16 and SKN-1 TFs may work in parallel or in sequence with ELT-2 during recovery from acute *S. enterica* infection [117], but the DAF-16 and SKN-1 TFs do not play a role in the context of acute *P. aeruginosa* infection [118]. In the search for an ELT-2 partner protein, RPT-6, a component of the 19S proteasome subunit, was found to interact with ELT-2 to control the expression of immune response genes (Figure 1C). Moreover, the mediation of ELT-2 through a proteasome was not only restricted to regulate immunity, but was also essential in the recovery phase from bacterial infection [119]. Besides its response to infections, ELT-2 is essential in the response to some non-infection types of stress, such as osmotic and hypoxic stress and high levels of dietary zinc [120,121].

### 4.3. Potential Areas for Future Research

Future work should further explore the role of ELT-2 in immune regulation while focusing on important areas such as the interaction of ELT-2 with other known TFs that mediate the immune response. Moreover, an understanding of the conserved role of ELT-2 across species considering its role in recovery from infection is essential.

## 5. Overview of *C. elegans* Transcription Factors in Immune Regulation

In this review, we have briefly summarized the current knowledge of three TFs (DAF-16, ZIP-2, and ELT-2) to highlight the importance of these TFs as the sole regulators of innate immunity besides other well-known immune pathways (PMK-1, DBL-1, and FSHR-1). Nonetheless, other transcription factors belonging to nuclear hormone receptors (NHRs), homeobox, and basic helix–loop–helix (bHLH) families have also been reported to be involved in modulating innate immunity of *C. elegans*. NHRs family members, NHR-23 and NHR-25 are reported to regulate the immune response [122,123], while DAF-12, through *let-7*, negatively regulates the immune response by targeting SKN-1 [124]. Moreover, some NHRs have been found to link innate immunity with metabolism, such as NHR-49, which was found to induce fat utilization during infection, and NHR-8, which is crucial for cholesterol-mediated immunity [125,126]. This highlights the importance of NHR transcription factors in immune regulation, linking it with metabolic processes to maintain immune homeostasis. Another homeobox transcription factor has been reported to play a vital role in regulating the immune response, in addition to its well-known activity in development and cellular differentiation. Recently, VAB-3 TF, a member of the homeobox transcription factor family, was reported to play a role in regulating the immune response to oomycetes. Moreover, VAB-3 controls the expression of receptor tyrosine kinases OLD-1 and FLOR-1, which are crucial in mounting an immune response to oomycetes in the epidermis [127]. Furthermore, CEH-37 (OTX2 in mammals) is another important homeobox TF that regulates the expression of C-type lectin receptors, which are essential in recognizing oomycetes. CEH-37 is involved in neuro-immune crosstalk to enhance defense mechanisms in the epidermis against pathogens [128,129]. CEH-37 also regulates the conserved PMK-1/p38 immune pathway in the intestine by suppressing the activity of its negative regulator, VHP-1 [129]. Moreover, TF CEH-60 also regulates the activity of some immune effectors in response to pathogen stress. Additionally, UNC-62 is crucial to maintain immune and metabolic homeostasis under stress conditions to ensure effective defense against pathogens [130]. The transcription factor family bHLH contains HLH-30, which is reported to play a pivotal role in immunity by regulating ATG-2 expression under pathogen stress. Intriguingly, HLH-30 demonstrates the gender-specific regulation of immunity in *C. elegans*, with male animals exhibiting strong resistance to pathogen stress comparatively to hermaphrodites, highlighting the crucial role of TFs in mediating the immune response across gender boundaries [131]. Altogether, these findings demonstrate that transcription factors play a pivotal role in innate immune regulation, particularly in the nuclear hormone receptor, homeobox, and basic helix–loop–helix families.

## 6. Concluding Remarks and Future Prospects

**Figure 2:**
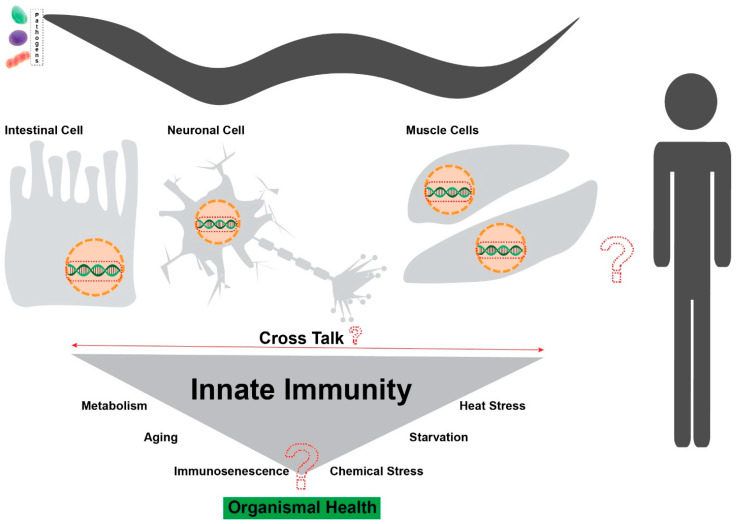
Illustrative model showing research gaps related to intercellular signaling, stress response (heat stress, starvation, and chemical stress), immunosenescence, metabolism, aging, and conserved function in humans. The question mark indicates areas for further exploration.

The knowledge shared in this review increases our understanding of the roles of TFs in regulating *C. elegans* immunity, either independently or by potentially contributing to other immune-specific signaling pathways in response to pathogens. Immune pathways solely controlled by TFs (DAF-16, ZIP-2, and ELT-2) have been discussed in detail. In addition, studies of a few members of the bZIP and WH-FORKHEAD TF families were shown to be highly important in the regulation of immune response genes to tackle infections. These findings underscore the importance of TFs in coordinating robust defenses against pathogenic threats. Additionally, this review highlights the signaling pathways regulated by TFs, which, to some extent, link innate immunity with other biological processes, such as aging, longevity, and non-pathogenic stress, illustrating the complexity in the relationship between the immune system and other biological processes (Figure 2). Importantly, these insights into *C. elegans* provide a basis for the evolutionarily conserved mechanisms that may occur in humans, given the conservation of TFs across families, which could lead to the development of novel ideas for clinical therapeutic strategies.

Innate immunity in worms still needs to be explored by considering how TFs are regulated by different virulent and bacterial components, as well as determining the co-regulators (proteins) of TFs that are involved in the stimulation or inhibition of immune responses. Moreover, the TFs discussed in this work primarily regulate immune responses to pathogens in the intestine. However, the mechanisms by which transcription factors modulate immune responses across different tissue types or participate in cross-tissue communication remain largely unexplored. Recent studies have begun to shed light on these complex interactions. For instance, a recent publication demonstrated that gamma-aminobutyric acid (GABA) inhibits TFs in the intestine to enhance gut immunity [43]. Additionally, the scope of the crosstalk between TFs, both within and across tissue types, remains unclear (Figure 2). For example, CEH-37 regulates the immune response through the PMK-1 immune pathway, a pathway that is also required for SKN-1’s function in response to stress. While CEH-37 and SKN-1 may act in response to distinct stress conditions, their dependence on PMK-1 suggests that there is a potential interaction or convergence in immune signaling. Understanding whether such crosstalk is a generalized mechanism across different pathogens could provide critical insights into immune homeostasis. Similarly, the bZIP transcription factors ZIP-10 [81] and ZIP-11 [80] both regulate immune responses to PA14 infection through the PMK-1 pathway. Interestingly, ZIP-10’s activity is modulated by GABA signaling, while ZIP-11 acts as a key activator of the same immune pathway. However, their potential functional interplay remains uninvestigated. Given that ZIP-10 suppresses, and ZIP-11 activates PMK-1 signaling, further exploration of their regulatory balance could reveal how TFs regulate the immune response to maintain homeostasis. Moreover, as discussed, TFs’ capacity to control the expression of immune response genes and their activation is primarily governed by signaling pathways; their expression and activity may also be influenced by chromatin modifications and promoter accessibility [132]. Recent studies indicate that epigenetic mechanisms, such as 6 mA DNA methylation, contribute to immune gene regulation [133,134]. However, whether these mechanisms influence the regulation of the specific TFs discussed in this review remains unknown. Exploring this connection could provide valuable insights into additional layers of transcriptional control in the immune system.

Besides molecular mechanisms of innate immune defense, *C. elegans* utilizes multiple approaches to defend itself against microbial threats. This includes behavioral avoidance, where nematodes actively elude potentially harmful microorganisms. However, our knowledge of TFs in the context of avoiding pathogen stress is still limited. Understanding the role of TFs in behavioral responses could offer new insights into the integration of behavior and immunity. In addition, we have briefly illustrated some immune pathway regulators (DAF-16, ZIP-2, and ELT-2) that act individually in the immune response, but their interactions or combined effects have not been extensively explored in terms of their molecular mechanisms or immune response. It would be worth exploring whether they work synergistically (to boost the immune response) or antagonistically (to inhibit other types of activity), as this could reveal a novel regulatory network that deepens our understanding of the immune response.

In conclusion, although *C. elegans*’ transcription factors have been proven to play a significant role in the immune response, either directly or indirectly, this review represents an underexplored area in the study of immunity, stress regulation, and potentially even behavioral responses through transcription factors. The diverse roles of TFs, both within and beyond immune response pathways, require further attention to fully explore their potential as components of health and disease (Figure 2). Subsequent studies should not only focus on their immune functions, but also identify their roles in maintaining cellular, organismal, and behavioral homeostasis. This may lead to the identification of novel dimensions of immune function, behavior, and longevity.

## 7. Challenges and Methodological Advances in Studying Transcription Factors in *C. elegans* Immune Regulation

Studying the global gene regulation imposed by TFs in *C. elegans* presents several challenges, including the complexity of the regulatory networks, the functional redundancy among TFs, and the temporal and spatial specificity of TFs’ activity. To address these challenges in the context of innate immunity research, advanced techniques such as ChIP-seq and RNA-seq can be utilized to map TFs’ binding sites and gene expression changes. CRISPR-Cas9 technology would enable the precise genetic manipulation of immune response TFs, while tissue-specific expression systems would allow for more targeted analyses. The integration of computational approaches would facilitate the prediction of TFs’ binding sites and regulatory networks. By addressing these challenges, researchers can enhance their understanding of the ways in which specific TFs, such as DAF-16, ZIP-2, HSF-1, ELT-2, and SKN-1, regulate the innate immune response in *C. elegans*. This approach may lead to the identification of novel immune-related TFs and their targets, advancing our knowledge of innate immunity regulation in *C. elegans*.

Several methods have been employed to study the TFs involved in immune regulation in *C. elegans*, each with its strengths and limitations. RNA-seq is a key approach to identifying which TFs’ expression patterns change after pathogen stress. However, it is limited by its inability to distinguish direct from indirect effects. To overcome this limitation, the creation of mutant strains of specific TFs could help to validate their precise roles. Forward genetics can be used to identify novel TFs involved in immune regulation but may fail to detect redundant factors. The creation of double mutants allows for the exploration of TFs’ interactions and redundancies, but the interpretation of complex phenotypes can be challenging. Tissue-specific knockdown using CRISPR-Cas9 can offer insights into TFs’ functions in specific tissue types, but off-target effects are a concern. Alternative strategies to address these limitations include ChIP-seq to identify direct TF binding sites. Single-cell RNA-seq can provide higher-resolution insights into TF activity across different cell types. Lastly, comparative studies with other organisms could help to identify conserved TFs’ functions in immune regulation.

## Figures and Tables

**Table 1:** Microbial species that are pathogenic to both humans and *C. elegans*.

Pathogen	Type of Microbe	Pathogenic to Humans and *C. elegans*?	Reference
*Pseudomonas aeruginosa*	Gram-negative bacterium	Yes	[33]
*Enterococcus faecaelis*	Gram-positive bacterium	Yes	[21]
*Burkholderia cenocepacia*	Gram-negative bacterium	Yes	[34]
*Cryptococcus neoformans*	Fungus	Yes	[27]
*Serratia marcescens*	Gram-negative bacterium	Yes	[35]
*Staphylococcus aureus*	Gram-positive bacterium	Yes	[26]
*Salmonella enterica*	Gram-negative bacterium	Yes	[36]

**Table 2:** Microarray and RNA sequencing studies focusing on differentially expressed TFs.

Pathogen	Exposure Time (Hours)	Differentially Expressed TFs	Upregulated TFs	Downregulated TFs	Reference
*S. aureus*	8	08	04	04	[41]
*S. marcescens*	24	57	27	30	[42]
*E. faecalis*	24	50	22	28	[42]
*P. luminescens*	24	62	30	32	[42]
*P. aeruginosa* (RNA Seq)	24	233	104	129	[43]

## Data Availability

No new data were generated for this review.
